# A randomized, double-blind, placebo-controlled study of a GHSR blocker in people with alcohol use disorder

**DOI:** 10.1172/jci.insight.182331

**Published:** 2024-12-20

**Authors:** Monica L. Faulkner, Mehdi Farokhnia, Mary R. Lee, Lisa Farinelli, Brittney D. Browning, Kelly Abshire, Allison M. Daurio, Vikas Munjal, Sara L. Deschaine, Selim R. Boukabara, Christopher Fortney, Garrick Sherman, Melanie Schwandt, Fatemeh Akhlaghi, Reza Momenan, Thomas J. Ross, Susan Persky, Lorenzo Leggio

**Affiliations:** 1 Clinical Psychoneuroendocrinology and Neuropsychopharmacology Section, Translational Addiction Medicine Branch, National Institute on Drug Abuse (NIDA) Intramural Research Program, National Institute on Alcohol Abuse and Alcoholism (NIAAA) Division of Intramural Clinical and Biological Research, NIH, Baltimore and Bethesda, Maryland, USA.; 2 Department of Mental Health, Johns Hopkins Bloomberg School of Public Health, Baltimore, Maryland, USA.; 3 Immersive Simulation Program, Social and Behavioral Research Branch, National Human Genome Research Institute (NHGRI), NIH, Bethesda, Maryland, USA.; 4 Office of the Clinical Director, NIDA, Intramural Research Program, NIH, Baltimore, Maryland, USA.; 5 Office of the Clinical Director, NIAAA Division of Intramural Clinical and Biological Research, NIH, Bethesda, Maryland, USA.; 6 Clinical Pharmacokinetics Research Laboratory, Department of Biomedical and Pharmaceutical Sciences, College of Pharmacy, University of Rhode Island, Kingston, Rhode Island, USA.; 7 Clinical NeuroImaging Research Core, NIAAA, NIH, Bethesda, Maryland, USA.; 8 Neuroimaging Core, NIDA Intramural Research Program, NIH, Baltimore, Maryland, USA.; 9 Center for Alcohol and Addiction Studies, Department of Behavioral and Social Sciences, Brown University, Providence, Rhode Island, USA.; 10 Division of Addiction Medicine, Department of Medicine, School of Medicine, Johns Hopkins University, Baltimore, Maryland, USA.; 11 Department of Neuroscience, Georgetown University Medical Center, Washington DC, USA.

**Keywords:** Clinical trials, Addiction

## Abstract

**BACKGROUND:**

Studies have demonstrated the role of ghrelin in alcohol-related behaviors and consumption. Blockade of the growth hormone secretagogue receptor (GHSR), which is the ghrelin receptor, has been shown to decrease alcohol drinking and reward-related behaviors across several animal models. We previously conducted a human study testing a GHSR inverse agonist/competitive antagonist, PF-5190457, in individuals who are heavy drinkers and showed its safety when coadministered with alcohol. Here, we conducted a phase IIa experimental medicine study in patients with alcohol use disorder (AUD) to investigate the effects of PF-5190457 on alcohol- and food-related outcomes.

**METHODS:**

Forty-two individuals with AUD (*n* = 29 completers) participated in a randomized, double-blind, placebo-controlled study where they received PF-5190457 100mg b.i.d. (or placebo) in 2 counterbalanced, within-subject stages. Participants completed an alcohol cue-reactivity (CR) experiment in a bar-like laboratory and a virtual food choice experiment in a cafeteria-like virtual reality (VR) environment. A subset of participants (*n* = 12) performed a CR task during a brain functional MRI (fMRI) experiment.

**RESULTS:**

PF-5190457 did not reduce cue-elicited alcohol craving. PF-5190457 reduced virtual calories selected (*P* = 0.04) in the VR environment. PF-5190457 did not influence neural activation during CR task in the fMRI experiment.

**CONCLUSION:**

This study provides human evidence of the role of GHSR blockade in behaviors related to food selection and highlights the need for future investigations into targeting the ghrelin system in AUD.

**TRIAL REGISTRATION:**

ClinicalTrials.gov (accession no. NCT02707055).

**FUNDING:**

NIDA and NIAAA ZIA-DA000635; National Center for Advancing Translational Sciences UH2/UH3-TR000963.

## Abstract 2

This study provides some of the first human evidence of the role of ghrelin receptor (GHSR) blockade in behaviors related to attention to alcohol cues and food selection.

## Introduction

Alcohol use disorder (AUD) and excessive alcohol drinking is a leading cause of preventable morbidity and mortality worldwide (1, 2). Treatment for AUD often includes a combination of psychological, social, and pharmacologic interventions, and effectiveness is highly variable across individuals (3, 4). There are currently only 3 Food and Drug Administration–approved (FDA-approved) medications for AUD, disulfiram, naltrexone, and acamprosate; hence, there is a critical need to develop additional novel effective treatments for AUD (3, 4).

The ghrelin system has recently gained attention as a potential pharmacotherapeutic target for AUD. The 28–amino acid peptide acyl-ghrelin (here referred to as ghrelin) (5) is an endogenous agonist for the growth hormone secretagogue receptor (GHSR), a G-protein coupled receptor which when activated induces growth hormone (GH) release from the pituitary (6) and stimulates appetite via changes in the activity of both orexigenic and anorexigenic neurons (7), among several other functions (8–10).Preclinical studies have demonstrated ghrelin’s role in the modulation of reward processing and alcohol use (11, 12). For example, ghrelin administration into the laterodorsal tegmental (LDTg) and ventral tegmental areas (VTA) increased extracellular concentrations of accumbal dopamine in mice (13), and these effects, as well as alcohol intake and alcohol-induced conditioned place preference, were attenuated in GHSR-KO mice (14). Similarly, alcohol binge-like drinking was reduced in GHSR-KO rats, compared with WT controls (15). Importantly, ghrelin administered into the lateral hypothalamus or paraventricular nucleus did not reduce alcohol drinking, suggesting that the neural regions regulating the effects of ghrelin on alcohol intake do not overlap completely with those that regulate the effects of ghrelin on appetite and food intake (14). Ghrelin has also been shown to modulate the central amygdala function, a brain region involved in stress regulation and alcohol consumption (16). Furthermore, ghrelin has been implicated in the modulation of negative emotional processing and stress-related neural pathways via the hypothalamic/pituitary/adrenal (HPA) axis and amygdala (17). Systemic and central administration of GHSR antagonists has been repeatedly shown to decrease alcohol intake and alcohol-seeking behaviors in different rodent models and across several independent laboratories (11–18).

Clinical studies have also demonstrated a relationship between alcohol use and ghrelin. Acute alcohol administration reduces circulating endogenous ghrelin levels (19–26), while chronic alcohol consumption appears to increase ghrelin levels (27, 28). Baseline ghrelin levels are positively correlated with alcohol craving (29–32) and may predict relapse to alcohol drinking (29). In 2 double-blind, placebo-controlled, experimental medicine studies, our team showed that i.v. ghrelin administration increased cue-elicited alcohol craving (33) and i.v. alcohol self-administration (34) in heavy-drinking individuals with AUD. The latter study also found that, during an alcohol/food incentive delay task, i.v. ghrelin administration increased alcohol-related neural activation in the amygdala, increased food-related neural activation in the nucleus accumbens, and decreased food-related neural activation in the medial orbitofrontal cortex (mOFC) (34). Together, these studies highlight the role of the ghrelin system in alcohol-related behaviors and support research into the ghrelin system as a potential therapeutic target for AUD.

PF-5190457 is a selective GHSR inverse agonist/competitive antagonist that inhibits constitutive activity of GHSR and competitively blocks its activation by ghrelin (35, 36). PF-5190457 was originally developed for the treatment of type 2 diabetes mellitus, and the manufacturer moved forward with its development up to a phase Ia first-in-human study, which showed its safety and tolerability in healthy individuals; common side effects were mild sedation and sleepiness (37). To our knowledge, PF-5190457 is the only GHSR inverse agonist/competitive antagonist that has advanced to clinical stages of medication development. In a previous study, we showed the safety, tolerability, and lack of drug-alcohol interactions with PF-5190457 in both rats and humans. We also performed a preliminary assessment of the effects of PF-519047 on craving in a bar-like laboratory. Findings suggest that PF-5190457, compared with placebo, reduced alcohol and food cue–elicited craving, as well as attention to alcohol cues (38).

Here, we followed up on our previous work and conducted a phase IIa human laboratory study to test the hypothesis that PF-5190457 may reduce alcohol and food cue–elicited craving and neural activation, attention to cues, and food choice behavior in individuals with AUD.

## Results

### Study sample.

**Figure 1:**
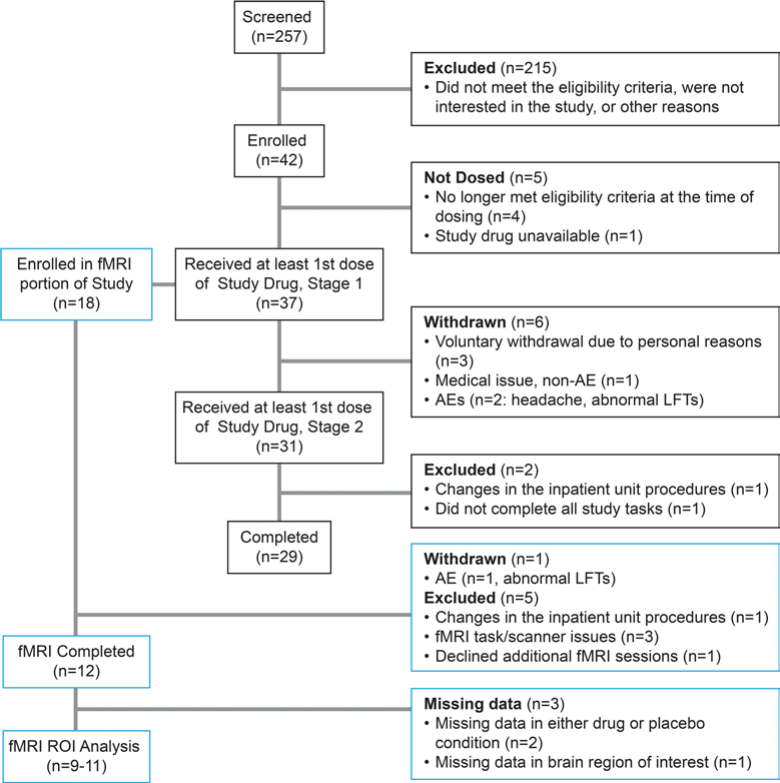
CONSORT flow diagram of screening, enrollment, and completion of the study. AE, adverse events; fMRI, functional MRI; LFTs, liver function tests; ROI, region of interest.

The study was terminated early due to the COVID-19 pandemic and temporary closure of our inpatient unit, resulting in a smaller sample than planned (29 participants completed the study, among whom 12 completed the fMRI; Figure 1). Participants were seeking treatment for AUD and had been residing in the NIAAA inpatient unit at the NIH Clinical Center (Bethesda, Maryland, USA) for an average (SD) of 34.4 (31.46) days from admission to enrollment into this study. Characteristics of the study sample are presented in Table 1. Participants were administered PF-5190457 and placebo for an average of 6.0 (1.6) and 5.6 (1.4) days, respectively.

### Cue reactivity (CR) in a bar-like laboratory.

**Figure 2:**
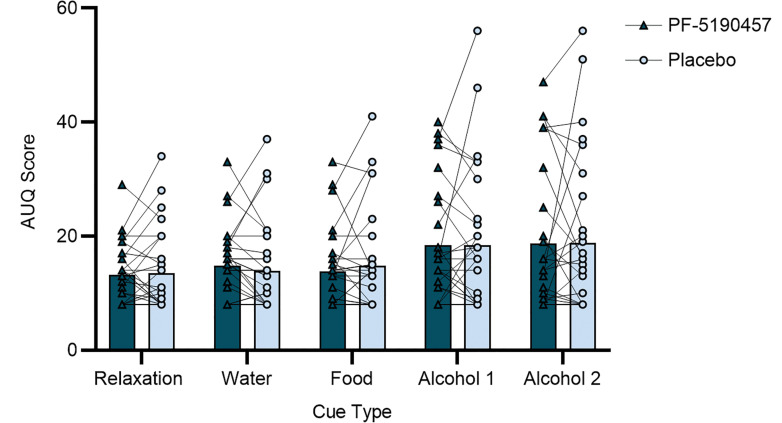
Alcohol craving during cue-reactivity (CR) in a bar-like laboratory. There was no main effect of PF-5190457 on alcohol cue–elicited craving as measured by the Alcohol Urge Questionnaire (AUQ) (Drug, 18.57 [11.93], versus Placebo, 18.59 [13.12]; F_1,39.9_ = 0.07, *P* = 0.80). There was no Drug × Time Point interaction (F_4,124_ = 1.17, *P* = 0.33). There was a significant effect of Time Point on alcohol cue–elicited craving (F_4,111_ = 9.00, *P* < 0.0001) driven by increased AUQ score after exposure to alcohol cues.

Participants were presented with water, food, and alcohol cues across individual trials in a bar-like laboratory. One participant became emotionally distressed, did not complete one of the 2 CR sessions, and was not included in the analysis. PF-5190457, compared with placebo, had no significant effect on alcohol cue–elicited craving (Alcohol Urge Questionnaire [AUQ] score) (see Methods); there was no Drug main effect (F_1,39.3_ = 0.07, *P* = 0.80), nor a Drug × Time Point interaction (F_4,124_ = 1.17, *P* = 0.33). There was a significant effect of Time Point which was driven by the presentation of alcohol cues (F_4,111_ = 9.00, *P* < 0.0001) (Figure 2). Based on these findings, we also compared alcohol craving on study Day 1 (prior to any drug administration) to craving during the CR experiment. Higher baseline AUQ scores on study Day 1 were associated with greater reduction in AUQ scores following exposure to alcohol cues under PF-5190457 compared with placebo, but the correlation did not reach statistical significance (*r* = 0.293, *P* = 0.13; Supplemental Figure 1; supplemental material available online with this article; https://doi.org/10.1172/jci.insight.182331DS1).

There was a Drug × Time Point interaction for the Alcohol Attention Scale (AAS) item 5 “*How much did you try to stop thinking about drinking when the alcoholic drink was presented?*” (F_1,46.2_ = 5.94, *P* = 0.02) (Supplemental Figure 2) (see Methods). However, post hoc tests of this interaction did not reveal a significant effect of Drug × Time Point-1 (first presentation of alcohol cue; Tukey-Kramer *P* = 0.41), Drug × Time Point-2 (second presentation of alcohol cue; Tukey-Kramer *P* = 0.97), or Drug × Time Point (combined Time Points 1 and 2, Tukey-Kramer *P* = 0.55). There was also no main effect of Drug (F_1,16.9_ = 0.38, *P* = 0.55) or Time Point (F_1,19.7_ = 1.08, *P* = 0.31) on AAS item 5. There was a significant effect of Time Point (F_1,30.8_ = 4.88, *P* = 0.03), but no Drug or Drug × Time Point effect, on the AAS item 2 “*How much did you pay attention to the smell of the alcoholic drink when it was presented?*” (see Methods) participants reported paying less attention to the smell of the alcohol drink during the second than the first alcohol trial. There were no significant findings on other AAS items (Supplemental Table 1).

There was a significant effect of Time Point (F_4,102_ = 13.59, *P* < 0.0001) but no significant effect of the Drug (F_1,35_ = 0.22, *P* = 0.65) nor a Drug × Time Point (F_4,130_ = 1.11, *P* = 0.35) interaction on food cue–elicited craving during the CR procedure as measured by the General Food Craving Questionnaire-State (GFCQ-S); this Time Point effect was driven by the presentation of the food cues.

Additional assessments of alcohol craving (AUQ), food craving (GFCQ-S), and mood state (profile of mood states; POMS) (see Methods) were taken prior to the administration of the drug and approximately 45 minutes after the completion of the CR procedure in the bar laboratory. These analyses revealed only an effect of Time Point on GFCQ-S (F_1,26.8_ = 13.40, *P* = 0.001), indicating increased food craving following the CR experiment, with no other Drug or Drug × Time Point interaction effects (Supplemental Table 2).

### Cafeteria-like virtual reality (VR) buffet.

**Figure 3:**
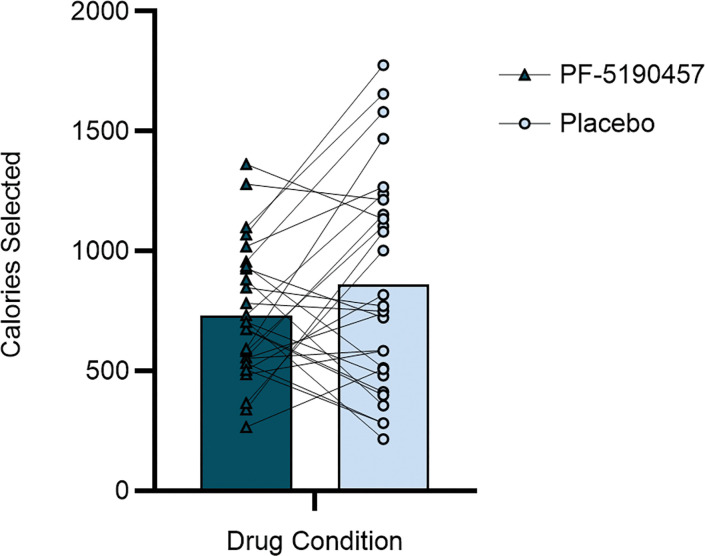
Total calories selected during cafeteria-like virtual reality (VR) buffet. There was a significant Drug effect, with fewer calories selected under PF-5190457 than Placebo (731.5 [275.7] versus 860.4 [448.6] calories; F_1,28.4_ = 4.50, *P* = 0.04).

Under PF-5190457, participants selected significantly fewer virtual calories compared with the placebo condition (731.5 [275.7] versus 860.4 [448.6] calories; F_1,28.4_ = 4.50, *P* = 0.04) (Figure 3).

Predrug to post-VR analyses showed only an effect of Time Point on GFCQ-S (F_1,35.1_ = 20.75, *P* < 0.0001), indicating increased food craving following the VR experiment, with no other Drug or Drug × Time Point interaction effects on food craving, alcohol craving, or mood states. (Supplemental Table 2).

### Functional MRI (fMRI) CR.

Out of 18 participants who were enrolled in the fMRI portion of the study, 1 was excluded from the analysis due to changes in the inpatient unit procedures, 1 was withdrawn from the study due to an adverse event (AE), and 4 others were excluded due to missing imaging data due to scanner imaging issues, scanner task issues, or declining further fMRI sessions. Under the drug condition, 2 participants’ data were missing, 1 in the erotic condition and 1 in the food and alcohol conditions. Under the placebo condition, 2 participants’ data were missing in the erotic condition. Only participants with data under both drug and placebo conditions were included in the analysis, resulting in 12 participants, 10 in the erotic condition, 10 in the nonerotic condition, and 11 in the food and alcohol conditions (some nonoverlapping participants across conditions). There was no significant effect of the study drug on neural activation during the alcohol, food, erotic, or nonerotic trials. Paired-sample 2-tailed *t* tests revealed a significant increase in neural activation in the left amygdala and right nucleus accumbens during only the Alcohol – Control contrast; however, these findings did not survive corrections for multiple comparisons (Table 2).

Analyses of food craving measured predrug to post-fMRI showed a significant main effect of the Drug (F_1,9.7_ = 5.17, *P* = 0.04) and main effect of Time Point (F_1,9.3_ = 12.19, *P* = 0.01). Food craving increased after fMRI, and participants reported lower food craving under the PF-5190457 condition; however, there was no Drug × Time Point interaction. There were no other Drug, Time Point, or Drug × Time Point interaction effects on alcohol craving or mood states.

### AEs.

AEs were assessed daily (Supplemental Data File 1), and all reported AEs were determined to be mild to moderate. There were no serious AEs during the study. Fisher’s exact test revealed no significant effect of drug condition on the total number of AEs (*P* = 1.00), nor the frequency of individuals AEs (Supplemental Table 3). There was no significant main effect of Drug (F_1,34.9_ = 0.56, *P* = 0.46), Time Point (F_2,56.7_ = 0.40, *P* = 0.67), nor Drug × Time Point interaction (F_2,74.4_ = 0.03, *P* = 0.97) on sleepiness (Stanford Sleepiness Scale [SSS]) or anhedonia (Snaith-Hamilton Pleasure Scale [SHAPS]) (see Methods) (Drug [F_1,61.1_ = 2.21, *P* = 0.14]; Time Point [F_1,32.6_ = 0.60, *P* = 0.45]; Drug × Time Point [F_1,26.2_ = 0.76, *P* = 0.39]). Analysis of the change in EKG variables from the day before drug dosing to the day after CR (the day after dosing) revealed a main effect of Drug on QT interval (F_1,55.8_ = 4.40, *P* = 0.04), but not on QT_C_B (corrected, Bazett formula) interval (F_1,54.1_ = 1.59, *P* = 0.21), nor QT_C_F (corrected, Fridericia formula) interval (F_1,54.1_ = 0.00, *P* = 0.97). There were no effects of PF-5190457 versus placebo on weight (F_1,27_ = 0.33, *P* = 0.60), waist circumference (F_1,51.9_ = 1.17, *P* = 0.28), blood pressure (diastolic [F_1,76.9_ = 0.01, *P* = 0.91]; systolic [F_1,65.3_ = 0.32, *P* = 0.58]), heart rate (F_1,59.5_ = 0.07, *P* = 0.80), or blood glucose levels (F_1,80.6_ = 0.34, *P* = 0.56). There were no significant effects of the Drug or Drug × Time Point on liver tests as measured by alanine transaminase levels (F_1,54.5_ = 0.44, *P* *=* 0.51, and F_1,54.5_ = 0.06, *P* = 0.80, respectively) and aspartate transferase levels (F_1,53.9_ = 0.07, *P* *=* 0.79, and F_1,53.9_ = 2.40, *P* = 0.13, respectively) or renal function as measured by creatinine levels (F_1,54.1_ = 1.36, *P* = 0.25, and F_1,54.1_ = 0.08, *P* = 0.78, respectively).

## Discussion

We investigated the effects of a GHSR inverse agonist/competitive antagonist, PF-5190457, on reactivity and attention to alcohol- and food-related cues in a bar-like laboratory and a brain fMRI experiment and assessed the effects on food choice behavior in a cafeteria-like VR buffet. We did not find an effect of PF-5190457 on our primary outcome, cue-elicited craving in the bar-like laboratory. We found that PF-5190457 significantly decreased the number of calories selected during the cafeteria-like VR buffet. PF-5190457 had no effect on neural activation in response to alcohol, food, or sexually erotic visual cues. We saw an overall effect of time on food craving, which we attribute to increase in general hunger due to study procedures being prior to lunch.

In terms of safety measures, we did not see differences in the frequency or severity of AEs between PF-5190457 and placebo. However, consistent with our previous phase Ib study (38), we found that PF-5190457 significantly increased QT interval, although this difference was not significant for the corrected QT intervals (QT_C_F and QT_C_B). From a clinical standpoint, like our phase Ib study (38), QT, QT_C_F, and QT_C_B intervals were never equal to or above 500 ms. Therefore, it is unlikely that the statistically significant change in QT interval was of clinical significance.

Our current findings do not indicate that PF-5190457 affects alcohol cue–elicited craving in individuals seeking AUD treatment. This finding is contrary to our previous smaller study that suggested reduced cue-elicited craving for alcohol under PF-5190457 in nontreatment seeking heavy-drinking individuals (38). One important difference is that, while this study was a double-blind placebo-controlled and counterbalanced study, our previous phase Ib study followed a fixed-order design for safety reasons; hence, we could not completely disentangle drug effects versus nonspecific time effects. In our previous phase Ib study, participants were nontreatment seeking, heavy-drinking individuals (at least 14 or 21 drinks per week on average for women or men, respectively). Our current study sample greatly differs from that, as participants were treatment-seeking individuals with AUD and had been receiving inpatient treatment for several weeks prior to enrollment in this study. Therefore, variations across the 2 samples, especially differences in baseline days of abstinence, as well as differences between treatment seekers, nontreatment seekers, and heavy drinkers without a diagnosis of AUD, as we and others have previously reported (39–42), may account, at least in part, for the differences between our phase Ib study (38) and the present study. Throughout the course of inpatient AUD treatment and related complete abstinence from alcohol, patients in this study may have begun to develop ways to cope with alcohol craving, and this could have influenced our ability to detect an effect of PF-5190457. We tried to account for these coping skills and possible habituation to the bar-like environment with the within-subjects counterbalanced design of the experiment. However, participants under both drug conditions had an average AUQ score of 18 when exposed to alcohol in the bar-like laboratory, while in our previous study, participants had an average AUQ score of 40 under placebo and 30 under PF-5190457. These differences suggest that the current study sample may have had lower cue-elicited craving in general, resulting in a floor effect. The correlation of baseline craving and alcohol cue–elicited craving during the CR procedure was not statistically significant. However, higher AUQ scores at baseline were associated with greater effects of PF-5190457 on cue-elicited craving in this study (Supplemental Figure 1). Assessing the effect of PF-5190457 at various stages of AUD treatment may aide in a better understanding of its effects on cue-elicited craving and other alcohol-related outcomes. In addition, CR can be characterized as both a state and trait measure (43). Some individuals, known as cue nonreactors, may not exhibit significantly increased urge or craving to consume alcohol after cue exposure (44). We did not screen participants based on their baseline alcohol CR. It would be important in future studies to explore individual differences in CR at baseline to better assess the effects of investigational pharmacotherapies on this outcome, as CR has been widely established as a clinically relevant outcome for alcohol use and AUD treatment (45–47).

During the CR experiment, we found a significant Drug × Time Point effect of PF-5190457 interaction on attention to alcohol, a finding consistent with our previous phase Ib study (38). However, post hoc tests were not significant; hence, there is no conclusive evidence of a significant effect of the drug on attention to alcohol. We also found a significant effect of time on reducing attention to the smell of the alcohol drink during the CR procedure, suggesting that attention to the smell of the cue diminished over time.

PF-5190457 had a significant effect on food choice during the virtual buffet and reduced the total calories selected. This finding is consistent with ghrelin’s known role in the regulation of feeding behavior (10, 48–52). Moreover, preclinical studies have demonstrated that GHSR blockade reduces food intake and weight (53–55), as does KO of GHSR in mice (56) and rats (57); in the latter study, we found that reduction in high-fat, diet-related weight gain was only present in male GHSR-KO rodents. We also demonstrated that central administration of PF-5190457 attenuated ghrelin-induced food intake in male, but not in female mice. However, we have also previously reported that PF-5190457 dose dependently inhibits food intake in fed (non–food-restricted) and fasting (food-withheld overnight) rats with exploratory analyses indicating no sex differences (58). Of note, the studies summarized above looked at GHSR-KO, PF-5190457, or both in the context of food seeking and diet-induced obesity without any alcohol-related experiments. Here, by contrast, we examined PF-5190457’s effects on VR-based food choices in the context of an alcohol study in individuals with AUD.

These results support the role of PF-5190457 on feeding behaviors in people with AUD and represent what we believe is the first human evidence that GHSR blockade does influence food-related outcomes. Although more research is needed, several studies have demonstrated strong correlations between food choices in a VR experiment and food choices in a real-world setting (59–61), suggesting that the effects of PF-5190457 on virtual food selection may extend to real-world food choice behaviors. In alcohol-related studies, GHSR blockade has also been shown to decrease alcohol and food preference and intake in rodents (62–67).

Interestingly, PF-5190457 did not have a significant effect on self-reported food craving (by GFCQ-S) after the VR procedure, suggesting that the drug may affect eating behavior via mechanisms unrelated to food craving. Given that we tested food choice but not alcohol choice (or alcohol self-administration) and given that we did not find an effect of PF-5190457 on either alcohol craving or food craving, it is conceivable that, contrary to our original hypothesis, PF-5190457’s effects on food and/or alcohol-related outcomes are not mediated by craving-related mechanisms. Together, the present results suggest that the potential role of PF-5190457 in AUD, if further investigated, should be studied by human experiments that involve alcohol choice, self-administration and drinking, and binge-like drinking, regardless of alcohol craving. This direction is consistent not only with the present results but also with our recent mouse studies indicating that PF-5190457 reduces binge-like alcohol drinking (68, 69). Furthermore, it is also noteworthy that a recent metaregression suggests low predictive utility of cue-induced alcohol craving alone in predicting clinical outcomes, including abstinence and heavy drinking in randomized controlled trials (70). PF-5190457 may not be effective in AUD, or it may belong to that group of medications for which cue-induced alcohol craving alone is not the best predictor of efficacy. On the other side, the present study provides more promising findings on the effects of PF-5190457 on feeding-related outcomes. This potential role of PF-5190457 should be further investigated in eating behaviors and obesity, an approach that is also in line with our recent pharmacological and transgenic rat work on GHSR and obesity (57, 58).

We have previously shown that i.v. ghrelin administration increased alcohol-related neural activation in the amygdala, decreased food-related neural activation in the mOFC, and increased food-related neural activation in nucleus accumbens (34). Furthermore, Koopman and colleagues investigated the role of endogenous ghrelin in AUD and found that alcohol craving ratings were mediated by alcohol cue–induced neural activation in the mesolimbic pathway of detoxified individuals with AUD (71). In the current study, we found that PF-5190457 had no significant effect on neural activation in our regions of interests, including amygdala, ventral striatum (VS), insula, nucleus accumbens, and dorsal anterior cingulate cortex (dACC). The modified version of the Amygdala Reactivity Task (see Methods) used in this study did appear to activate the amygdala, as expected, in the alcohol and erotic conditions (Supplemental Figure 3, A–D). GHSR blockade in preclinical studies has been shown to reduce neural activation in mesolimbic neural regions, motivation for rewards, and alcohol consumption (64–69). In line with the low craving levels in the bar-like laboratory, we speculate that, in this small sample of recently detoxified and treatment-seeking individuals with AUD, confounding factors — e.g., treatment status (recently detoxified and currently in treatment) — may have contributed to these null neuroimaging results. Interestingly, a recent study of healthy individuals without AUD found that i.v. ghrelin administration significantly attenuated neural activation in the striatum in anticipation of losing a reward and delay discounting (72, 73), contrasting the reward motivating role of ghrelin in mesolimbic dopamine signaling seen in numerous preclinical investigations. Together these studies highlight the need to further investigate the relationship of ghrelin and reward-related outcomes to better understand neural mechanisms underlying the ghrelin-reward interactions in humans.

Another possibility is that the lack of PF-5190457’s effects on our neuroimaging outcomes reflects its limited ability to cross the blood-brain barrier, a question that will need future studies to be fully elucidated, given some inconsistent literature. PF-5190457 was developed as a compound with limited ability to reach the brain (“brain-impaired,” as defined in the original phase I human study conducted by Pfizer, given its limited central exposure; ref. 37). A previous study shows that PF-5190457 reduces alcohol preference in male mice during a 2-bottle free-choice procedure; however, this effect disappeared after capsaicin-induced vagal deafferentation, suggesting that PF-5190457’s effects on alcohol drinking behaviors are peripherally mediated (74). PF-5190457’s limited ability to reach the brain could also explain the lack of effects on cue-elicited alcohol craving. We have previously shown that, after systemic administration, PF-5190457 is detected and quantified in the rat brain, which, however, does not necessarily mean it is functionally engaged to central GHSRs (38). Moreover, in a recent mouse study, we showed that the endogenous GHSR antagonist liver-expressed antimicrobial peptide-2 (LEAP2) reduces alcohol drinking when administered centrally but not systemically, while systemic PF-5190457 reduced alcohol drinking, suggesting that direct or indirect engagement of central GHSRs may be necessary to affect alcohol drinking (68). Given that this was an inpatient experimental medicine study, future human studies are needed to test whether PF-5190457 does reach the brain in a pharmacologically meaningful way; whether, consistent with the recent mouse studies (68, 69), PF-5190457 may reduce alcohol drinking; and, if so, whether these effects are mediated via peripheral, central, or combined mechanisms.

A limitation of this study was its small sample size. The study was terminated early due to the COVID-19 pandemic resulting in enrollment of fewer individuals than originally planned. This had a particular effect on the fMRI portion of the study. Another limitation of the study was the experimental setting following inpatient treatment for AUD. The experimental bar-like setting was likely not able to evoke strong craving in this sample, as they knew they were still residing in a controlled treatment environment. Notwithstanding its limitations, this study also holds several strengths, including the within-subject design and the well-controlled experimental medicine design. The study is one of the very few studies testing a GHSR inverse agonist/competitive antagonist in humans, following the first-in-human study conducted by the manufacturer (37) and our own phase Ib study in people with AUD (38). Of note, to the best of our knowledge, this is the first clinical study testing a GHSR inverse agonist/competitive antagonist in humans via several experimental procedures, including behavioral (CR and VR) and neuroimaging (fMRI) outcomes.

It is important to note that, given the inpatient settings of this study, we only assessed experimental outcomes related to cue-elicited craving, alcohol attention, VR-based food choice, and brain fMRI outcomes. We did not assess whether PF-5190457 may influence real-world alcohol drinking, a question that may only be addressed in humans via future outpatient clinical studies.

This study provides important information regarding GHSR blockade via the compound PF-5190457 in individuals with AUD, including its tolerability and safety, as well as its potential pharmacological effects on alcohol- and food-related outcomes. Our findings highlight the need for future research investigating the ghrelin system as a potential pharmacotherapeutic target for AUD, eating disorders, and/or obesity.

## Methods

### Sex as a biological variable

Both biological male and female participants were included in this study.

### Study design

**Figure 4:**
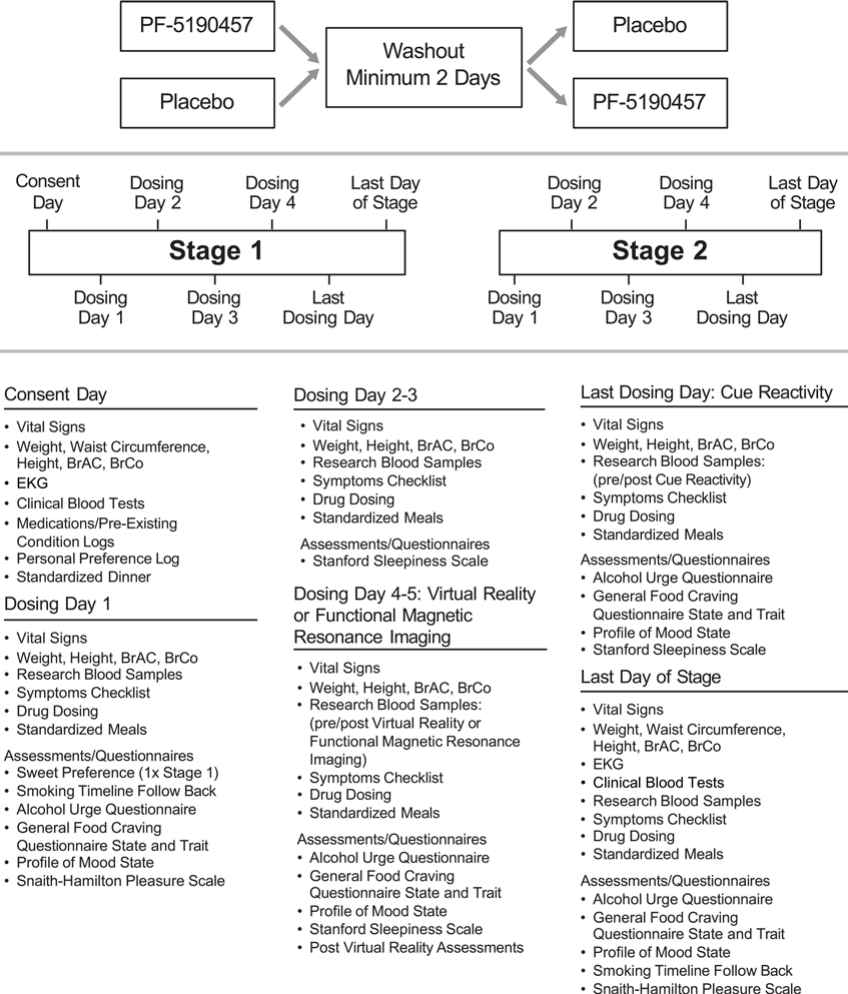
Study outline with timeline of study assessments. BrAC, breath alcohol concentration; BrCo, breath carbon monoxide concentration.

The study was a randomized, within-subject, counterbalanced, double-blind, placebo-controlled, phase IIa clinical trial conducted at the NIH Clinical Center. Participants were enrolled in the study typically a few weeks after enrollment in the NIAAA screening and natural history protocol, under which they received medically supervised alcohol detoxification in an inpatient unit. Participants remained hospitalized throughout the present study and continued to participate in standard-of-care AUD treatment activities — e.g., general medical care and individual and/or group therapy sessions. The study included 2 counterbalanced stages (Stage 1 and Stage 2), in which participants received PF-5190457 100 mg b.i.d. up to steady state, requiring 3 days of drug dosing or placebo, and completed identical study procedures during each stage. Baseline assessments (Figure 4) were collected before the first dose of the study drug or placebo at Stage 1. Once during each stage and after the study drug reached steady state, participants underwent assessments measuring CR craving (primary outcome) and attention to alcohol cues in a bar-like laboratory and a cafeteria-like VR buffet experiment. A brain fMRI was also performed on a subset of participants who satisfied additional MRI-specific eligibility criteria. Each stage included a minimum of 3 days of drug dosing only (Days 1–3), 1 VR day with drug dosing (Day 4 or 5), 1 optional brain fMRI day with drug dosing (Days 4 or 5), 1 CR day with morning only drug dosing (Day 5 or 6), and 1 postprocedure day (no dosing). The end of Stage 1 was followed by a minimum of 2 washout days, to allow complete elimination of the study drug. Then, Stage 2 took place (same as Stage 1), followed by study discharge. The order of the procedure days was identical for each stage, and the CR was always the last experiment, while VR and fMRI (the latter if done) could alternate. All 3 experimental procedures (bar-like CR, cafeteria-like VR, and fMRI) started at approximately 12:30 p.m., approximately 30 minutes after drug administration.

On drug-dosing days, participants were provided standardized meals at 9 a.m., 1:30 p.m., and 6 p.m., with the 1:30 p.m. meal always being provided after the completion of CR, VR, or fMRI. Questionnaires were administered around 10 a.m., followed by blood collection. Additional drug dosing days could be added to maximize feasibility and minimize scheduling issues, with a maximum of 14 days per stage (Figure 4).

### Participants

Forty-two (29 males, 13 females) individuals were enrolled in the study (Figure 1). Participants were recruited through advertisements and referrals for participation in the NIAAA screening and natural history protocol, under which individuals seeking treatment for AUD receive inpatient clinical care, including treatment for acute alcohol withdrawal and standard behavioral support while screened for participation in NIAAA clinical protocols. Participants were 18–70 years old with a current diagnosis of AUD, defined as meeting at least 2 of 11 symptoms on the Diagnostic and Statistical Manual of Mental Disorders, Fifth Edition (DSM-5) criteria on the Structured Clinical Interview for DSM Disorders (SCID) (75). All participants were medically stable and had a negative urine test for amphetamines, benzodiazepines, cannabinoids, cocaine metabolites, and opioids before enrollment. The study inclusion and exclusion criteria are listed in Supplemental Data File 2.

To standardize caloric intake within and across participants during the study, all participants received standardized meals provided by the NIH Clinical Center Nutrition Department. Standardized individual menus were determined by estimating caloric intake using the Mifflin-St Jeor equation with standard activity factor of 1.5 (aimed to allow ± 100 kcals) and controlling for macronutrients at the day level: each day was 50% carbohydrate, 20% protein, 30% fat (aimed to allow ± 5% difference for the overall day) (76). Menus were consistent between conditions unless a participant strongly requested a change in specific food items. Standardized meals were not provided during the washout period (Supplemental Data File 3).

### Study drug

PF-5190457 is an orally bioavailable GHSR inverse agonist/competitive antagonist with a half-life of approximately 6 hours (37, 38, 77). The drug was provided in-kind to the study team by Pfizer via a National Center for Advancing Translational Sciences (NCATS) Drug Development Partnership Program. In our previous phase Ib study (38), we tested 2 doses of PF-5190457 (50 mg b.i.d. and 100 mg b.i.d.), and both were determined to be safe and tolerable. Therefore, for this study, we selected the higher dose (100 mg b.i.d.). PF-5190457 was administered twice a day at 12 p.m. and 12 a.m. for the duration of the study. Given its half-life, steady state was assumed in all participants after at least 3 days of dosing.

### Study procedures

#### CR in a bar-like laboratory.

CR craving and attention to alcohol cues were assessed in a bar-like laboratory (Supplemental Figure 4), a procedure like our previous phase Ib study (38). Participants were exposed to visual, tactile, olfactory, and proprioceptive stimuli/cues associated with their preferred alcohol-containing beverage and food in a bar-like environment. The CR procedure started approximately 30 minutes after drug administration, around 12:30 p.m. Participants underwent 5 consecutive trials (relaxation, water, food, alcohol 1, and alcohol 2; the latter was a second exposure to the same type of alcohol beverage) and completed questionnaires to measure their alcohol (AUQ) and food (GFCQ-S) craving after each trial. AUQ is an 8-item self-administrated questionnaire that assesses current drinking urges and alcohol craving (78). GFCQ-S is a 15-item self-administered questionnaire that assesses current food craving (79). The AAS was also administered after the alcohol trials only. This self-administrated questionnaire consists of 5 items that assess how much attention was paid to the sight (item 1) and smell (item 2) of the alcohol, how much the participant thought about drinking the alcohol (item 3), how much the participant thought about other things rather than the drink (item 4), and how much they tried to stop thinking about drinking (item 5) (47, 80). During CR Day, participants completed questionnaires to assesses their alcohol craving (AUQ), food craving (GFCQ-S), and mood (POMS) prior to drug administration and approximately 45 minutes after the completion of the CR procedure, following research blood collection. POMS is a 65-item scale that assesses transient mood states by asking individuals to rate how much they are currently experiencing a mood state from “not at all” to “extremely” (81).

#### Cafeteria-like VR buffet.

Participants engaged with a cafeteria-like “virtual buffet” developed by the NHGRI Immersive Simulation Program (Supplemental Figure 4) (59, 82). The VR procedure started approximately 30 minutes after drug administration, around 12:30 p.m. Participants were instructed to choose foods and a nonalcohol beverage after completing a training session the same day. They wore a head mounted display, walked around the space to view available foods, and used a hand controller to make food selections. Foods and beverages were among those typically found at buffet-style restaurants, and the choices were composed of a range of nutrient profiles and calorie densities. Participants were instructed to choose as many and as much of the virtual food and 1 beverage during 1 trip to the buffet as they would normally choose for lunch. Once the virtual plate was full, participants were given the opportunity to go back to the buffet, select a second plate, and add additional virtual food. The buffet contained at least 2 options for each food category (main dish, vegetable, fruit, starch, dessert, and beverage). The experiment ended when participants indicated they were finished selecting their food and drink. Participants’ selections were digitally recorded, and their food choice behavior was assessed by calculating the total virtual calories selected. Calorie content was assessed by using the cubic volume of the virtual food/drink chosen, associating it with the most appropriate real-world weight of the item, and calculating the appropriate number of calories based on information contained in food nutrient databases. Similar to the CR Day, participants completed questionnaires to assesses their alcohol craving (AUQ), food craving (GFCQ-S), and mood (POMS) prior drug administration and approximately 45 minutes after the completion of the VR procedure, following research blood collection.

#### fMRI CR.

Participants underwent brain fMRI sessions at the Functional Magnetic Resonance Imaging Core Facility (FMRIF) at the NIH Clinical Center, approximately 30 minutes after drug administration, if they had neither contraindication for MRI nor colorblindness. Task-based blood oxygenation level-dependent (BOLD) imaging data were collected on a GE MR-750 3T scanner and 32-channel head coil (TR = 2 seconds, TE = 25 ms, flip angle = 70°, 36 slices; FoV = 21.6 mm; 72 × 72 mm voxels). A high-resolution T1-weighted coplanar image and a high-resolution magnetization-prepared rapid gradient-echo (MPRAGE) T1-weighted image was also acquired during imaging sessions. Imaging sessions, consisting of both structural and task-based scans, lasted approximately 1.5 hours. The primary fMRI outcome of interest was whether PF-5190457, compared with placebo, would reduce brain BOLD response during exposure to alcohol, food, and erotic cues. The addition of erotic cues (other than alcohol and food) was an exploratory outcome to investigate ghrelin signaling in sexually motivated reward–related behavior, based on research showing that ghrelin signaling is required for sexually motivated behavior in sexually naive male mice (83).

The block design fMRI task consisted of 2 counterbalanced runs of either alcohol- and food-related images or sexually explicit and nonsexually explicit images in a modified version of the Amygdala Reactivity Task (84). Each run consisted of 17 blocks, 8 blocks of appetitive cues (either food- and alcohol-related stimuli or sexual or nonsexual stimuli) and 9 blocks of geometric shape control stimuli (control condition). Each block contained 4 trials, and stimuli were displayed on the screen for a constant 5-second duration regardless of the speed of a participant’s response. Food and alcohol cues were compiled from online and laboratory databases, and sexually appetitive cues were 4 images of males and females that were most highly rated for positive valence in the Erotic/Romance category of the International Affective Picture System (IAPS), based on a previous study (85) (Supplemental Figure 5). Control cues for this task were taken from the NimStim picture set. Participants viewed 3 simultaneously presented stimuli in the formation of a triangle and selected 1 of the 2 choices of stimuli at the bottom that matched the target stimuli at the top of the triangle formation. Before and at end of the fMRI procedure, participants completed questionnaires to assess their alcohol craving (AUQ), food craving (GFCQ-S), and mood (POMS).

### Assessment of AEs

AEs were assessed by the study clinicians via a study-specific symptom checklist. If a participant endorsed “yes” to any symptom, further evaluation was done by the study physician and an AE was recorded. In addition to the symptom checklist, sleepiness was assessed on study Day 3 and on VR and CR procedure days, using the SSS (86). Anhedonia was assessed by the SHAPS (87) on Day 1 of the study and at the end of each stage. Additional measures of safety were assessed including (a) daily vital signs, breath alcohol concentration (BrAC) and weight, (b) EKG (twice during each stage), and (c) blood glucose levels via finger stick (3 times during each stage). Furthermore, blood clinical tests (electrolytes, liver, and renal function tests) were monitored at the beginning of Stage 1 before administration of the study drug, again at the end of Stage 1, and then twice during Stage 2 (before first drug administration and at the end of Stage 2) (Figure 4).

### Statistics

Power analysis conducted prior to study initiation using G*Power (88) determined that an effect size of *dz* = 0.4 would require a sample size of 41 participants for the primary outcome (bar-like cue-elicited craving). Statistical analyses were performed using SAS v.9.4 (SAS Institute) for behavioral data and AFNI (89) and MATLAB v.2022b for fMRI data. Forty-two participants were enrolled in the study; however, it was decided that 1 enrolled participant would be excluded from the statistical analysis due to changes in the inpatient unit procedures. Descriptive statistics were used to summarize demographic characteristics of the sample. Continuous and categorical variables are presented as mean (SD) and number (percent), respectively. Two-way repeated-measures ANOVA within a linear mixed model (SAS PROC MIXED) were used to assess the effects of PF-5190457 on alcohol cue–elicited craving and virtual food choices. For our primary outcome of cue-elicited craving measured by the AUQ, the analysis assessed craving during each CR trial (relaxation, water, food, and alcohol × 2) (Time Point main effect), a PF-5190457 versus placebo comparison (Drug main effect), as well as a Drug × Time Point interaction. We also assessed alcohol cue–elicited craving, food craving, and mood state before drug administration and after the procedure using similar models: predose and postprocedure (Time Point effect), PF-5190457 versus placebo (Drug main effect), and Drug × Time Point interaction. For our secondary outcome of food choices during the cafeteria-like VR buffet, the analysis compared the total number of calories selected under the PF-5190457 versus placebo condition. Similar to the CR experiment, alcohol craving, food craving, and mood were assessed before drug administration and after the procedure. The Kenward-Roger correction was used in all models using PROC MIXED, as the use of this correction is highly recommended in repeated measures models to account for bias of missing values. Post hoc comparisons were performed using Tukey tests. Measurements of additional factors such as sleepiness, anhedonia, waist circumference, weight, blood pressure, blood glucose levels, EKG, and liver and renal function were assessed several times during the study to monitor any changes associated with PF-5190457. We used 2-way repeated-measures ANOVA to evaluate any effects of the Drug or Drug × Time Point interactions on these factors as well. These analyses controlled for BMI, baseline blood ghrelin levels, age, sex, and drug condition order. Significance level was set at *P* < 0.05 (2-tailed) for all behavioral analyses.

For fMRI data, following standardized preprocessing using fMRIprep (Supplemental Data File 4), AFNI’s 3dREMLfit was used for generalized linear modeling to estimate individual effects of each contrast of interest (Alcohol – Control, Food – Control, Sexually Erotic – Control, Nonerotic – Control). AFNI’s 3dMEMA program was used for second-level group analysis comparing drug conditions (PF-5190457 versus placebo) and to determine mean condition specific regional responses, using a 2-tailed paired samples *t* tests, where *P* < 0.05 was considered significant. An a priori hypothesized region of interest (ROI) analysis was performed using the following anatomical ROI with known contributions to emotional processing, reward regulation, and CR as well as ghrelin signaling: amygdala (84, 90), VS (31, 71), insula (71, 91), nucleus accumbens (92), mOFC (34), and dACC (93). For each ROI, a 5 mm sphere was created around MNI coordinates and mean parameter estimates (β values) for each trial type (alcohol, food, erotic, nonerotic, and control) were extracted for both PF-5190457 and placebo conditions (Supplemental Table 4).

Due to the low frequency of AEs, Fisher’s exact tests were used to evaluate differences in the frequency of AEs between the PF-5190457 and placebo conditions.

### Study approval

The study was approved by the NIH IRB in Bethesda, Maryland, USA. This study was conducted under the FDA Investigational New Drug no. 119,365 (ClinicalTrials.gov; NCT02707055). A Data Safety and Monitoring Board (DSMB) provided independent reviews of the study. All participants provided written consent before enrollment and were compensated for their time and participation in the study.

### Data availability

Data used for Figures 2 and 3 and for Supplemental Figures 1, 2, and 3 are available in the Supporting Data Values file. All other data are available upon reasonable request from the corresponding author.

## Author contributions

LL conceptualized and developed the study scientific rationale and framework. MLF, MF, MRL, LF, FA, SP, and LL developed and implemented the clinical protocol. MLF, MF, MRL, LF, BDB, KA, AMD, VM, SLD, CF, RM, SP, and LL collected the data and/or assisted with and supervised data collection. MLF, MF, SRB, GS, MS, TJR, SP, and LL analyzed the data or assisted with and supervised data analyses. MRL and LL provided medical monitoring and oversight. FA, RM, SP, and LL provided funding or resources for this study. MLF, MF, and LL wrote the original draft of the manuscript. All authors reviewed and contributed to the manuscript and approved the final manuscript.

## Supplementary Material

Supplemental data

ICMJE disclosure forms

Supporting data values

## Figures and Tables

**Table 1:**
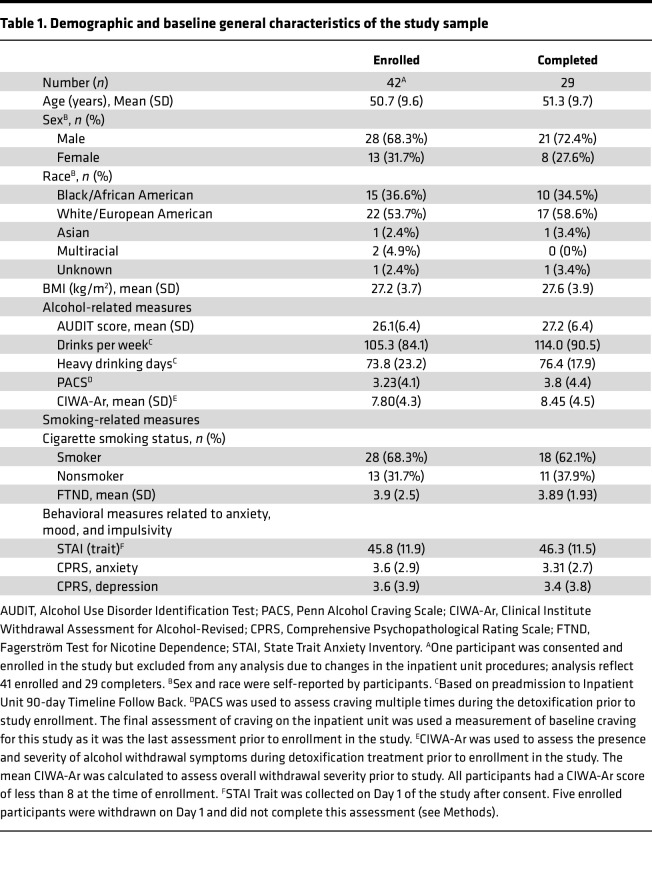
Demographic and baseline general characteristics of the study sample

**Table 2:**
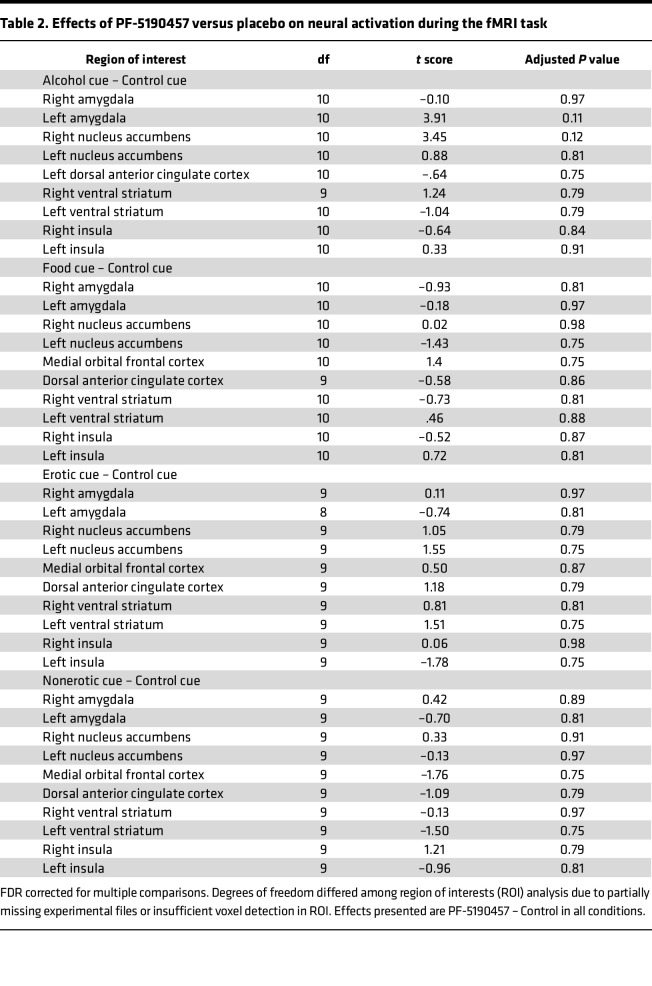
Effects of PF-5190457 versus placebo on neural activation during the fMRI task

## Version 1 12/20/2024

Electronic publication
