# Supplementary material for: A randomized, double-blind, placebo-controlled study of a GHSR blocker in people with alcohol use disorder
Source: JCI Insight. 2024 Dec 20;9(24):e182331. doi: 10.1172/jci.insight.182331 (PMC11665556; doi:10.1172/jci.insight.182331)
Supplement: Supplemental data [file jciinsight-9-182331-s214.pdf]

SUPPLEMENTARY MATERIAL

SUPPLEMENTAL DATA FILES

Supplemental Data File S1. Adverse Event Reporting Form

Adverse Event: (Diagnosis): ____

1. Status of Adverse Event:
   - ☐ New Start Date: ______________________

     Stop Date: ______________________ [Check here if ongoing: ☐]
   - ☐ Ongoing with change Describe: ___________________________________

     Date of worsening in Intensity: ___________________

2. Severity Grade:

☐ 1. Mild ☐ 2. Moderate ☐ 3. Severe ☐ 4. Life threatening

3. Relationship:
   - ☐ Unknown
   - ☐ Not Related
   - ☐ Remotely Related
   - ☐ Possibly Related
   - ☐ Probably Related
   - ☐ Definitely Related

4. Study Drug Action Taken:
   - ☐ None
   - ☐ Study Drug Withdrawn
   - ☐ Study Drug Dose Reduced

5. Treatment:
   - ☐ None
   - ☐ Prescribed by NIAAA Clinicians
   - ☐ Outside Referral
   - ☐ Emergency Room
   - ☐ Other

6. Outcome:
   - ☐ Recovered/Resolved
   - ☐ Recovering/Resolving
   - ☐ Not Recovered/Not Resolved
   - ☐ Recovered/Resolved with Sequelae
   - ☐ Fatal
   - ☐ Unknown

7. Event Type:
   - ☐ Non-Serious Expected
   - ☐ Non-Serious Unexpected
   - ☐ Serious Expected*
   - ☐ Serious Unexpected*

   [* For Serious Adverse Events, please submit SAE Form per protocol]

RECORDED BY: ________________REVIEWED BY: _____________ (Independent Safety Monitor (ISM))

____ (Medical Investigator or Designee)

Supplemental Data File S2. Inclusion and Exclusion Criteria

<u>Inclusion criteria</u>:

1. Males or females 18-70 years old (inclusive)
2. Current Alcohol Use Disorder by the Diagnostic and Statistical Manual of Mental Disorders, Fifth Edition (DSM-5) criteria
3. Most recent urine drug test negative for amphetamines, benzodiazepines, cannabinoids, cocaine, and opiates.
4. Most recent Clinical Institute Withdrawal Assessment for Alcohol-revised (CIWA-Ar) score less than or equal to 8.
5. Heart rate less than or equal to 100 on two separate measurements, both assessed after CIWA-Ar score is less than or equal to 8.
6. Female subjects must be of non-childbearing potential as defined by at least one of the following criteria:

   a) Females 45 – 70 years old, who are menopausal, defined as follows:

      i) Females who are 45 – 55 years old will be considered menopausal if they satisfy all the following three requirements during screening:

         1) they are in amenorrhea, defined as absence of menstruation for the previous 12 months;

         2) they have a negative urine pregnancy test; and

         3) they have a serum follicle-stimulating hormone (FSH) level within the laboratory's reference range for postmenopausal females.

      ii) Females who are 56 – 70 years old will be considered menopausal if they are in amenorrhea, defined before screening.

      OR

   b) Females 21 – 70 years old who have a documented hysterectomy and/or bilateral oophorectomy.

   All other female subjects, including those with tubal ligations and those that do not have a

documented hysterectomy, will be considered to be of childbearing potential.

7. Male subjects must use one of the following methods of contraception as defined by at least one of the following criteria;
   A. Abstinence
   B. A condom and one of the following:
      i. Vasectomy for more than 6 months.
      ii. The female partner must meet one of the following conditions:
         1) Has had a tubal ligation, hysterectomy, or bilateral oophorectomy.
         2) Is post-menopausal.
         3) Uses one of the following forms of contraception:
            a. Copper or hormonal containing intrauterine device
            b. Spermicidal foam/gel/film/cream/suppository
            c. Diaphragm with spermicide
            d. Oral contraceptive
            e. Injectable progesterone
            f. Subdermal implant

Exclusion criteria:

1. Lifetime clinical diagnosis of schizophrenia or bipolar disorder.
2. Electrocardiogram (EKG) with QTc greater than 450 msec as determined by the Fridericia formula.
3. A Body Mass Index (BMI) less than 18.5 kg/m^2, or a diagnosis of anorexia.
4. A BMI greater than or equal to 40 kg/m^2.
5. History of epilepsy and/or seizures (NOTE: individuals who have a history of alcohol withdrawal seizures may be in the study if they have been abstinent from alcohol for at least 2 weeks prior to consent and during that period of abstinence, there were no seizure episodes (otherwise, participant remains not eligible).

6. Most recent blood tests show creatinine greater than or equal to 2 mg/dL, aspartate aminotransferase (AST) or alanine aminotransferase (ALT) measurements 3 times larger than the upper normal limit, and a hemoglobin less than 10.5 g/dL.
7. Diabetes and/or treatment with any drug with glucose-lowering properties such as sulfonylurea, insulin, metformin, thiazolidinediones (TZD), Dipeptidyl peptidase-4 (DPP4) inhibitors, or Glucagon-like peptide-1(GLP-1) agonists (due to the glucose-lowering properties of PF-05190457 observed in healthy volunteers).
8. Exclusionary medications, supplements, and therapies:

   A. Naltrexone, acamprosate, alcohol dehydrogenase inhibitors, topiramate, gabapentin, ondansetron, benzodiazepines, barbiturates, baclofen, alpha-1 blockers, drugs that are known to prolong the QTc interval, hormone replacement therapy; medications and dietary/herbal supplements (e.g., St. John's wort) that interact with cytochrome P450 3A4. Patients who take these medications may be enrolled in the study only if the medication has been stopped for a period of at least 5 half-lives of the interacting medication before PF-5190457 administration.
9. Unable to pass a finger rub hearing test.
10. Vision is unable to be corrected to 20/100 on the Snellen Eye Chart.
11. Clinically significant history of motion or car sickness, or history of vestibular disorders.
12. Any other reason or clinical condition for which the principal investigator or the medical advisory investigator consider unsafe for a possible participant to participate in this study.

fMRI Exclusion Criteria

14. Have contraindications for brain functional Magnetic Resonance Imaging (fMRI), as determined by the National Institute on Alcohol Abuse and Alcoholism (NIAAA) MRI safety screening form.
15. Colorblindness (this would prevent the subject from completing the Stroop task) using the Ishihara Test for Color Deficiency, Concise Edition, 2014.

Supplemental Data File S3. Standardized Diet Menu Options

Participants were informed that they would receive standardized meals during the study. Participants were instructed to select up to 3 menu options in each meal category from the following list for the creation of their individualized study menu.

	**Breakfast**	**Lunch**	**Dinner**
**Option A**	Cheerios with Milk<br>Toast with Butter<br>Cottage Cheese<br>Pineapple	Roast Beef and Provolone Sandwich with Mayo, Lettuce, and Tomato on Multigrain Bread<br>Potato Chips<br>Grapes	Turkey<br>Mashed Potatoes and Gravy<br>Broccoli<br>Dinner Roll with Butter<br>Chocolate Chip Cookie
**Option B**	Pancakes with Butter and Syrup<br>Turkey Sausage<br>Peach Yogurt	Grilled Chicken and Provolone Wrap with Mayo, Lettuce, and Tomato<br>Apple<br>Pretzels	Grilled Chicken<br>Macaroni and Cheese<br>Broccoli<br>Grapes<br>Ginger ale
**Option C**	Scrambled Egg<br>Blueberry Muffin<br>Strawberry Yogurt<br>Peaches	Grilled Chicken Caesar Salad<br>Dinner Roll<br>Applesauce<br>Chocolate Chip Cookie	Beef Tender Roast<br>Baked Potato with Butter and Sour Cream<br>Green Beans<br>Angel Food Cake<br>Peaches
**Option D**	French Toast with Syrup<br>Scrambled Eggs with Cheddar Cheese	Turkey and Swiss Sandwich with Mayo, Mustard, Lettuce, and Tomato on Whole-Wheat Bread<br>Baby Carrots with Ranch Dressing<br>Pretzels<br>Tropical Fruit	Hamburger<br>French Fries with Ketchup
**Option E**	Pancakes with Butter and Syrup<br>Milk<br>Scrambled Eggs	Vegetable Quesadilla<br>Black Beans<br>Rice<br>Salsa<br>Orange	Baked Tilapia<br>Wild Rice Mix<br>Sautéed Spinach<br>Dinner Roll with Butter<br>Chocolate Pudding<br>Oreos
**Option F**	Omelet with Swiss Cheese, Spinach, Mushrooms and Onions<br>Whole-Wheat Toast with Butter and Strawberry Jelly	Turkey and Provolone Sandwich with Mayo and Mustard<br>Potato Chips<br>Grapes	Spaghetti with Marinara, Turkey Meatballs, and Parmesan Cheese<br>Dinner Roll<br>Side Salad with Italian Dressing

Supplemental Data File S4. Image Processing

Results included in this manuscript come from preprocessing performed using *fMRIPrep* 20.2.7 (@fmriprep1; @fmriprep2; RRID:SCR_016216), which is based on *Nipype* 1.7.0 (@nipype1; @nipype2; RRID:SCR_002502).

Anatomical data preprocessing

A total of 1 T1-weighted (T1w) images were found within the input BIDS dataset.The T1-weighted (T1w) image was corrected for intensity non-uniformity (INU) with `N4BiasFieldCorrection` [@n4], distributed with ANTs 2.3.3 [@ants, RRID:SCR_004757], and used as T1w-reference throughout the workflow.

The T1w-reference was then skull-stripped with a *Nipype* implementation of the `antsBrainExtraction.sh` workflow (from ANTs), using OASIS30ANTs as target template. Brain tissue segmentation of cerebrospinal fluid (CSF), white-matter (WM) and gray-matter (GM) was performed on the brain-extracted T1w using `fast` [FSL 5.0.9, RRID:SCR_002823, @fsl_fast]. Brain surfaces were reconstructed using `recon-all` [FreeSurfer 6.0.1, RRID:SCR_001847, @fs_reconall], and the brain mask estimated previously was refined with a custom variation of the method to reconcile ANTs-derived and FreeSurfer-derived segmentations of the cortical gray-matter of Mindboggle [RRID:SCR_002438, @mindboggle].

Volume-based spatial normalization to one standard space (MNI152NLin2009cAsym) was performed through nonlinear registration with `antsRegistration` (ANTs 2.3.3), using brain-extracted versions of both T1w reference and the T1w template. The following template was selected for spatial normalization: *ICBM 152 Nonlinear Asymmetrical template version 2009c* [@mni152nlin2009casym, RRID:SCR_008796; TemplateFlow ID: MNI152NLin2009cAsym],

Functional data preprocessing

For each of the 7 BOLD runs found per subject (across all tasks and sessions), the following preprocessing was performed. First, a reference volume and its skull-stripped version were generated using a custom methodology of *fMRIPrep*. Susceptibility distortion correction (SDC) was omitted. The BOLD reference was then co-registered to the T1w reference using

`bbregister` (FreeSurfer) which implements boundary-based registration [@bbr]. Co-registration was configured with six degrees of freedom. Head-motion parameters with respect to the BOLD reference (transformation matrices, and six corresponding rotation and translation parameters) are estimated before any spatiotemporal filtering using `mcflirt` [FSL 5.0.9, @mcflirt]. BOLD runs were slice-time corrected to 0.973s (0.5 of slice acquisition range 0s-1.95s) using `3dTshift` from AFNI 20160207 [@afni, RRID:SCR_005927].

The BOLD time-series (including slice-timing correction when applied) were resampled onto their original, native space by applying the transforms to correct for head-motion. These resampled BOLD time-series will be referred to as *preprocessed BOLD in original space*, or just *preprocessed BOLD*. The BOLD time-series were resampled into standard space, generating a *preprocessed BOLD run in MNI152NLin2009cAsym space*. First, a reference volume and its skull-stripped version were generated using a custom methodology of

fMRIPrep. Several confounding time-series were calculated based on the *preprocessed BOLD*: framewise displacement (FD), DVARS and three region-wise global signals. FD was computed using two formulations following Power (absolute sum of relative motions, @power_fd_dvars) and Jenkinson (relative root mean square displacement between affines, @mcflirt). FD and DVARS are calculated for each functional run, both using their implementations in *Nipype* [following the definitions by @power_fd_dvars]. The three global signals are extracted within the CSF, the WM, and the whole-brain masks.

Additionally, a set of physiological regressors were extracted to allow for component-based noise correction [*CompCor*, @compcor]. Principal components are estimated after high-pass filtering the *preprocessed BOLD* time-series (using a discrete cosine filter with 128s cut-off) for the two *CompCor* variants: temporal (tCompCor) and anatomical (aCompCor). tCompCor components are then calculated from the top 2% variable voxels within the brain mask. For aCompCor, three probabilistic masks (CSF, WM and combined CSF+WM) are generated in anatomical space. The implementation differs from that of Behzadi et al. (2007) in that instead of eroding the masks by 2 pixels on BOLD space, the aCompCor masks are subtracted a mask of pixels that likely contain a volume fraction of GM. This mask is obtained by dilating a GM mask extracted from the FreeSurfer's *aseg* segmentation, and it ensures components are not extracted from voxels containing a minimal fraction of GM.

Finally, these masks are resampled into BOLD space and binarized by thresholding at 0.99 (as in the original implementation). Components are also calculated separately within the WM and CSF masks. For each CompCor decomposition, the *k* components with the largest singular values are retained, such that the retained components' time series are sufficient to explain 50 percent of variance across the nuisance mask (CSF, WM, combined, or temporal). The remaining components are dropped from consideration. The head-motion estimates calculated in the correction step were also placed within the corresponding confounds file. The confound time series derived from head motion estimates and global signals were expanded with the inclusion of temporal derivatives and quadratic terms for each [@confounds_satterthwaite_2013]. Frames that exceeded a threshold of 0.5 mm FD or 1.5 standardised DVARS were annotated as motion outliers. All resamplings can be performed with *a single interpolation step* by composing all the pertinent transformations (i.e. head-motion transform matrices, susceptibility distortion correction when available, and co-registrations to anatomical and output spaces). Gridded (volumetric) resamplings were performed using `antsApplyTransforms` (ANTs), configured with Lanczos interpolation to minimize the smoothing effects of other kernels [@lanczos]. Non-gridded (surface) resamplings were performed using `mri_vol2surf`(FreeSurfer).

Many internal operations of *fMRIPrep* use *Nilearn* 0.6.2 [@nilearn, RRID:SCR_001362],

mostly within the functional processing workflow. For more details of the pipeline, see [the section corresponding to workflows in *fMRIPrep*'s documentation](https://fmriprep.readthedocs.io/en/latest/workflows.html "FMRIPrep's documentation").

Copyright Waiver

The above boilerplate text was automatically generated by fMRIPrep with the express intention that users should copy and paste this text into their manuscripts *unchanged*. It is released under the [CC0](https://creativecommons.org/publicdomain/zero/1.0/) license.

Supplemental Figure S1: Magnitude of the effect of PF-5190457 on alcohol cue-elicited craving compared to baseline alcohol craving

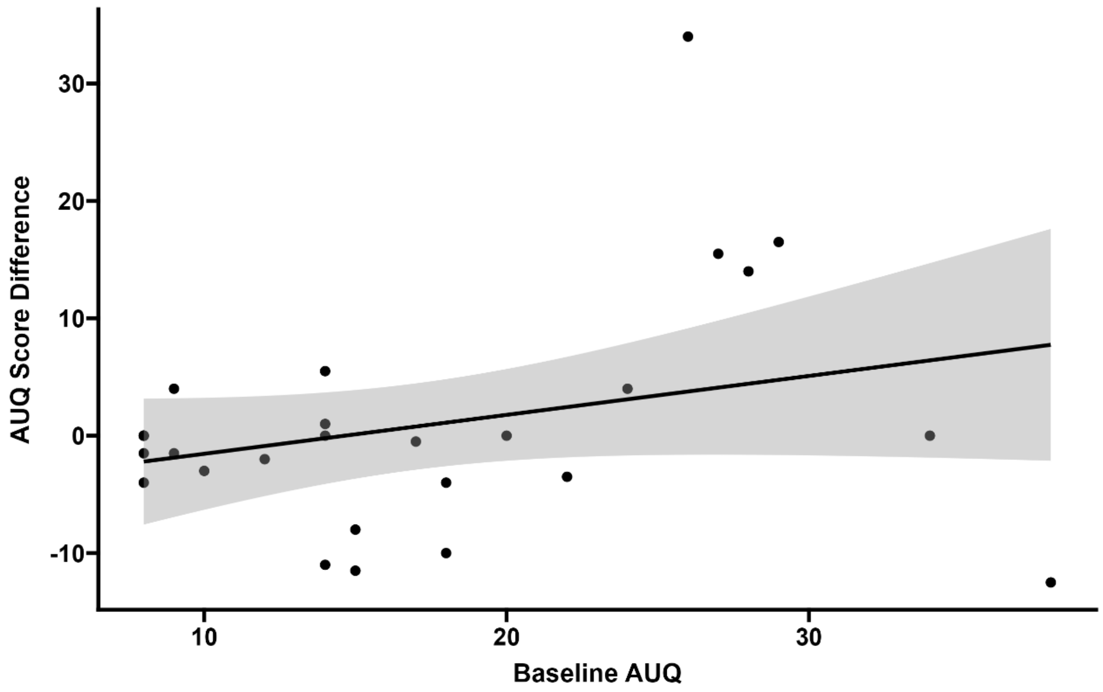

Legend to Figure S1. Baseline craving for alcohol was measured by the Alcohol Urge Questionnaire (AUQ) on Day 1 of the study prior to any drug administration. For both placebo and PF-5190457, average AUQ was calculated across the two trials. AUQ Score Difference was then calculated as the average under placebo minus the average under PF-5190457.

Pearson correlation: r = 0.293, p = 0.13.

Supplemental Figure S2. Attention to Alcohol Cues during Cue-Reactivity (CR) in a Bar-Like Laboratory

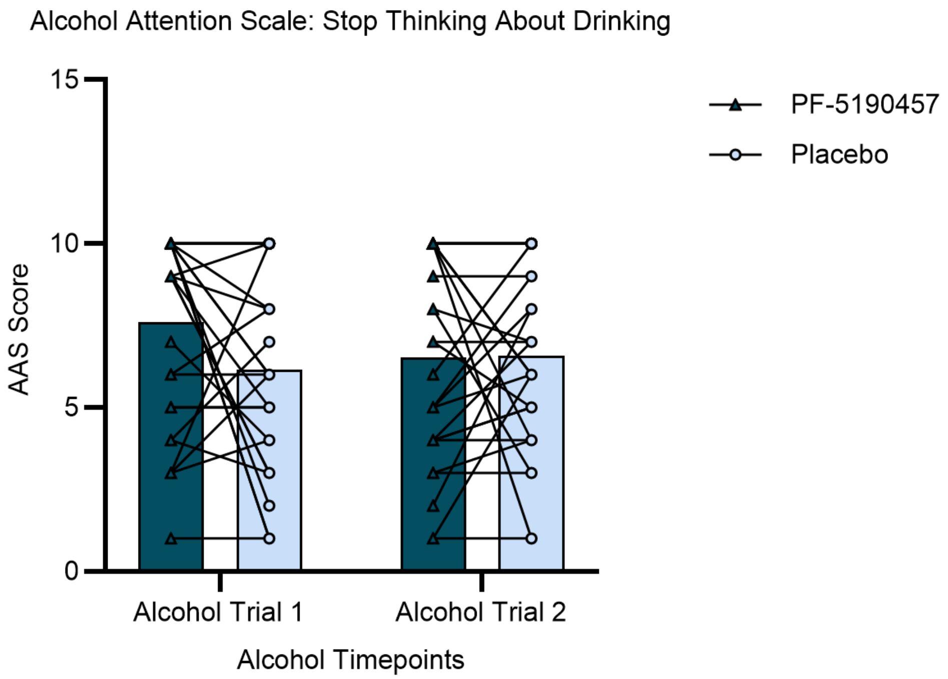

Legend to Figure S2. PF-5190457 x Timepoint interaction for the Alcohol Attention Scale (AAS) item 5 "*How much did you try to stop thinking about drinking when the alcoholic drink was presented?*" ($F_{1, 46.2} = 5.94$, $p = 0.02$). Post-hoc tests of this interaction did not reveal a significant effect of Drug × Timepoint-1 (Tukey-Kramer p=0.41), Drug × Timepoint-2 (Tukey-Kramer p=0.97), or Drug × Timepoint (combined Timepoints 1 and 2, Tukey-Kramer p=.55). [alcohol trial 1: Drug: 7.5 (2.76), Placebo: 7.25 (2.98); alcohol trial 2: Drug: 7.03 (2.98), Placebo 6.89 (3.24)].

Supplemental Figure S3: Amygdala Reactivity Task Activation Patterns during the fMRI

Figure S3-A

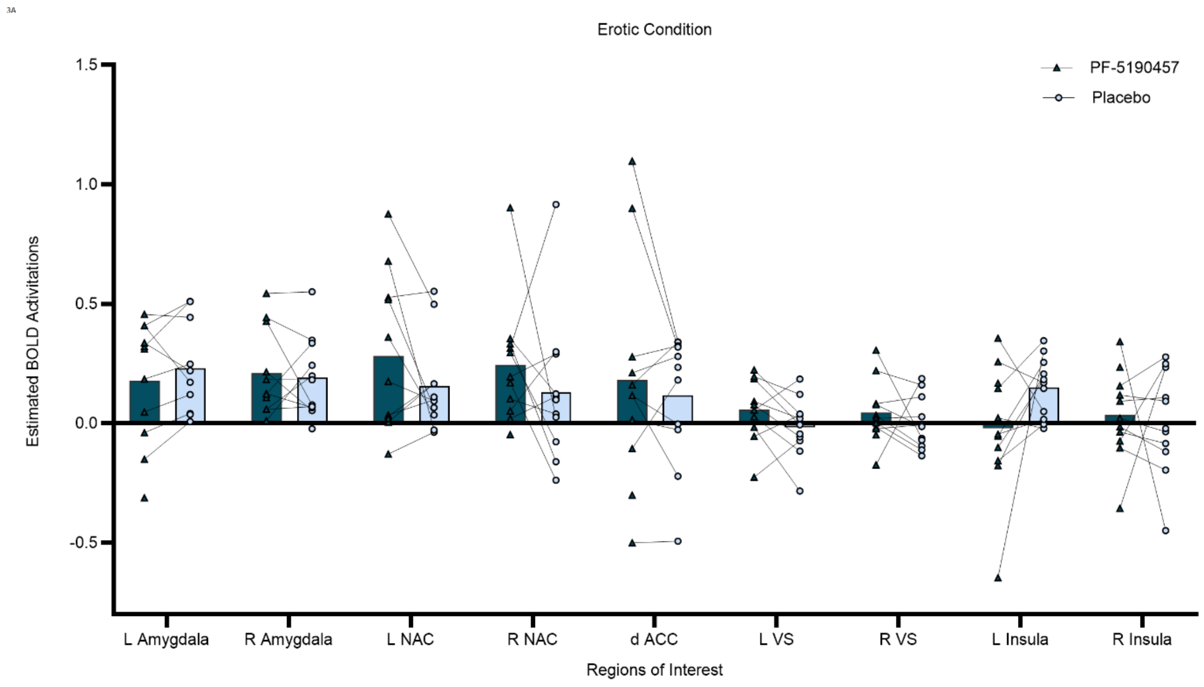

Figure S3-B

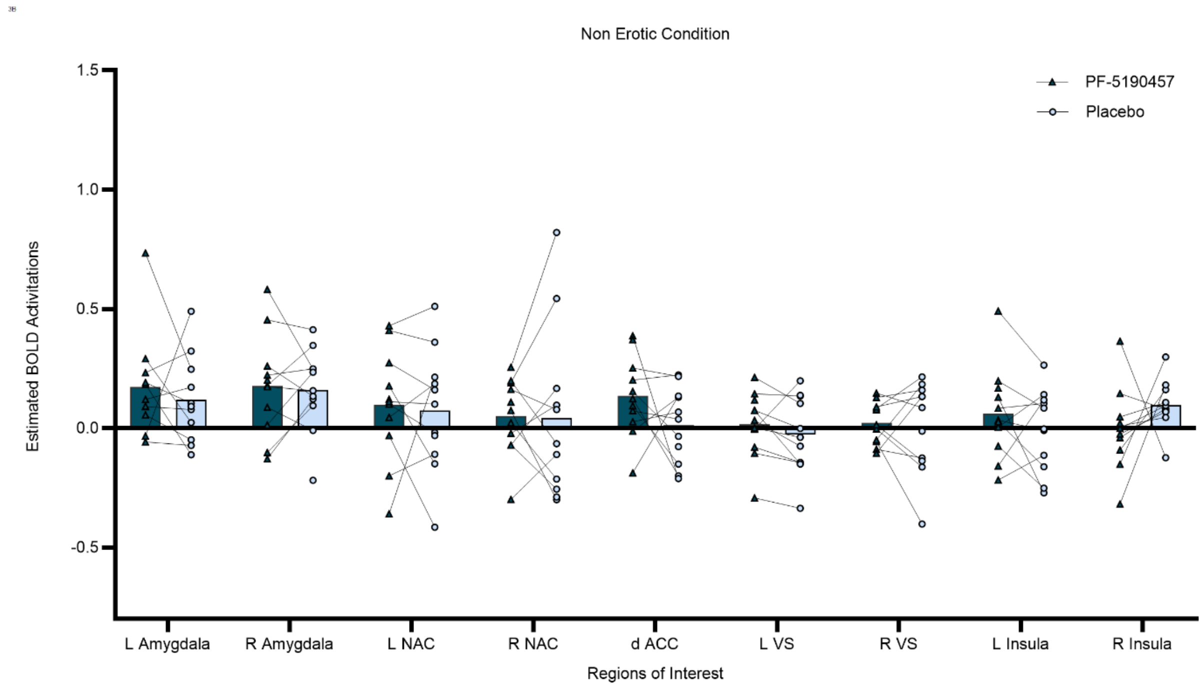

Figure S3-C

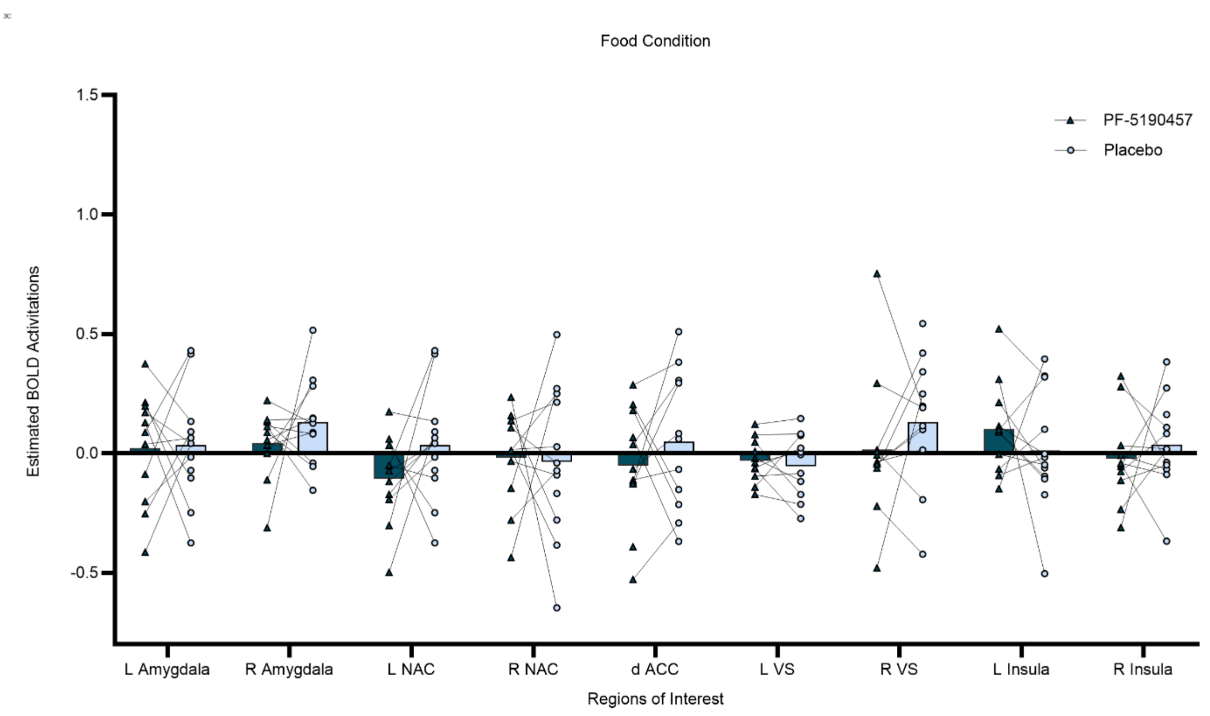

Figure S3-D

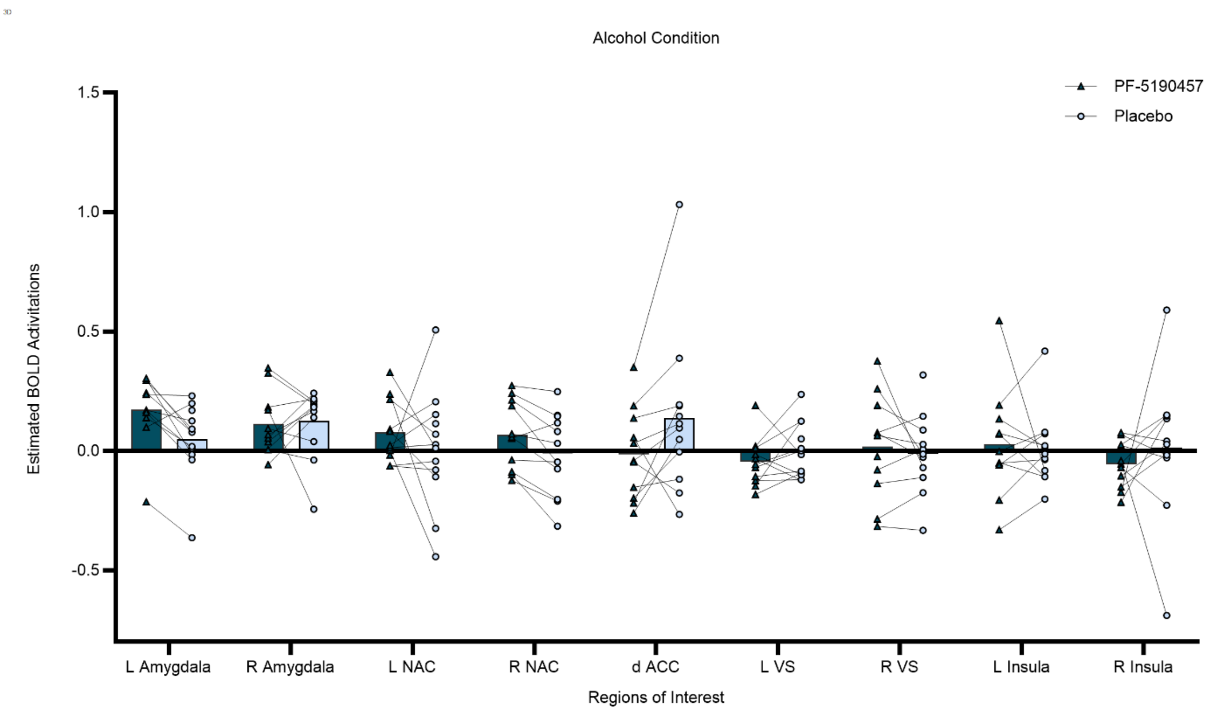

Legend to Figure S3. Activation Patterns for Regions of Interests (ROIS) during the Amygdala Reactivity Task during fMRI. L= Left, R= Right, NAC= Nucleus Accumbens, dACC= Dorsal Anterior Cingulate Cortex, VS= Ventral Striatum

S3-A. Erotic-Control condition in the PF-5190457 vs Placebo condition. N=10
S3-B. Non-Erotic-Control condition in the PF-5190457 vs Placebo condition. N=10
S3-C. Food-Control condition in the PF-5190457 vs Placebo condition. N=11
S3-D. Alcohol-Control condition in the PF-5190457 vs Placebo condition. N=11

Supplemental Figure S4. Bar-like Laboratory and Cafeteria-Like Virtual Reality Buffet

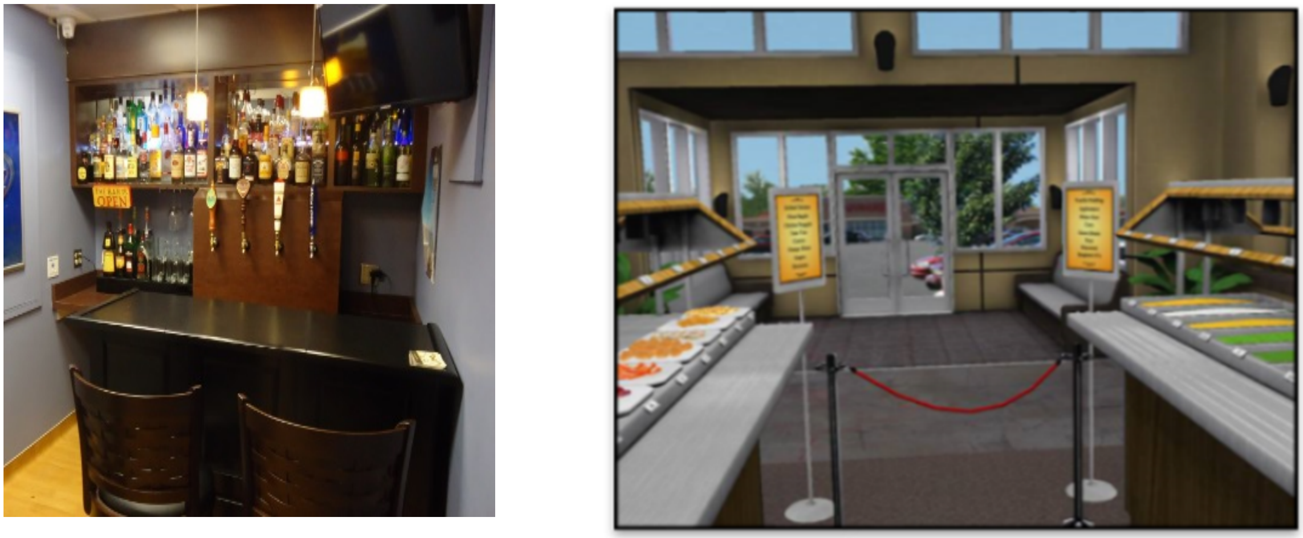

Legend to Figure S4. Image examples from the bar-like laboratory (left) and cafeteria-like virtual reality buffet (right)

Supplemental Figure S5: Schematic of fMRI Cue Reactivity

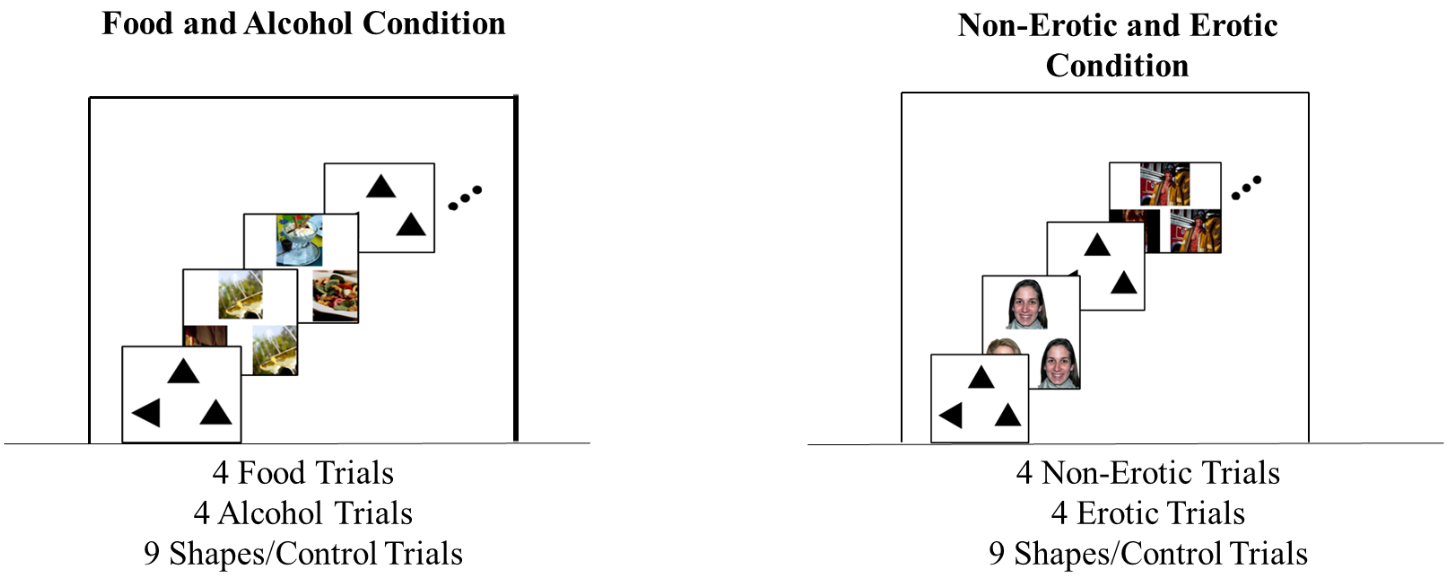

Legend to Figure S5. Visual depiction of the modified Cue Reactivity Task. Images of alcohol, food, sexually erotic, non-erotic, and shapes were presented in blocks during the functional Magnetic Resonance Imaging (fMRI) task. Food and alcohol images appeared in the same run; non-erotic and sexually erotic images appeared in a separate run. Images of shapes were used as control images. Participants were instructed to press the button for the bottom image that matched the top one.

SUPPLEMENTAL TABLES

Supplemental Table S1. Effects of the Drug (PF-5190457) versus Placebo on Alcohol Attention Scale (AAS) Items during the First and Second Alcohol Trial of the Bar-Like Cue-Reactivity.

AAS Item	Drug	Timepoint	Drug ×Timepoint
How much did you pay attention to the sight of the alcoholic drink when it was presented?	$F_{1,22.1}$= 0.04, *p*= 0.84	$F_{1,22.7}$= 2.10, *p*= 0.16	$F_{1,45.1}$= 0.02, p= 0.90
How much did you pay attention to the smell of the alcoholic drink when it was presented?	$F_{1,29.3}$=0.07, *p*= 0.80	**$F_{1,30.8}$= 4.88, *p*= 0.03**	$F_{1,39.7}$= 0.37, *p*= 0.54
How much did you think about drinking the alcoholic drink when it was presented?	$F_{1,18.5}$= 0.08, *p*= 0.78	$F_{1,19.4}$= 0.43, *p*= 0.52	$F_{1,46.4}$= 0.01, *p*= 0.94
How much did you think about other things rather than the drink when the alcoholic drink was presented?	$F_{1,23.3}$= 0.03, *p*= 0.87	$F_{1,23.5}$= 0.04, *p*= 0.83	$F_{1,44.4}$= 0.19, p= 0.66

Supplemental Table S2. Pre and Post Experiment Administration Behavioral Outcomes under the Drug (PF-5190457) versus Placebo.

	Drug	Timepoint	Drug × Timepoint
**Bar-like Cue Reactivity**			
AUQ	$F_{1,28.5}$= 0.63, p=0.44	$F_{1,26.9}$=0.35, p=0.56	$F_{1,40.1}$=1.27, p=0.27
GFCQ-S Total	$F_{1,27.1}$= 0.06, p= 0.82	$F_{1,26.8}$= 13.40, p=0.001	$F_{1,42.5}$= 0.72, p=0.40
POMS Total	$F_{1,17.6}$= 0.85, p= 0.37	$F_{1,19.6}$= 0.75, p= 0.40	$F_{1,46.7}$= 0.18, p= 0.67
**Cafeteria-like Virtual Reality Buffet**			
AUQ	$F_{1,29.6}$=0 .32, p=0.58	$F_{1,29.5}$=0.59, p=0.45	$F_{1,43.3}$=0.39, p=0.54
GFCQ-S Total	$F_{1,36.5}$=0.27, p=0.60	$F_{1,35.1}$=20.75, p<0.0001	$F_{1,33.4}$=1.63, p=0.21
POMS Total	$F_{1,17.7}$=0.14, p= 0.71	$F_{1,17.7}$= 0.02, p= 0.90	$F_{1,48.8}$ = 0.03, p= 0.87
**fMRI Cue Reactivity**			
AUQ	$F_{1,7.03}$=0.00, p=0.98	$F_{1,7.91}$=1.30, p=0.29	$F_{1,14.5}$=0.23, p=0.64
GFCQ-S Total	$F_{1,9.7}$=5.17, p=0.047	$F_{1,9.3}$=12.19, p=0.01	$F_{1,12.1}$=0.38, p=0.55
POMS Total	$F_{1,9.29}$= 3.61, p= 0.09	$F_{1,10.5}$=0.08, p= 0.78	$F_{1,12.6}$= 0.02, p= 0.89

Abbreviations: AUQ: Alcohol Urge Questionnaire; fMRI: functional Magnetic Resonance Imaging; GFCQ-S: General Food Craving Questionnaire–State; POMS: Profile of Mood States

Supplemental Table S3. Frequency of Adverse Events under the Drug (PF-5190457) versus Placebo

Adverse Events	Drug N	Placebo N	Fisher's Exact Test *P*
**Abdominal Discomfort/Pain**	1	1	1
**Ankle Swelling**	1	0	1
**Anxiety / Distress**	1	0	1
**Bloating/Flatulence**	4	4	1
**Constipation**	2	0	0.49
**Cough**	0	1	1
**Dental Pain**	1	0	1
**Depressive Mood**	0	1	1
**Diarrhea**	1	0	1
**Drowsiness/Fatigue/Lethargy/Sleepiness/Somnolence/Tiredness**	9	8	1
**Elevated Mood/Euphoria**	1	0	1
**Elevated Serum Creatinine**	0	1	1
**Elevated Serum Liver Enzymes**	1	1	1
**Headache / Pain**	4	3	1
**Hunger / Increased Appetite**	3	3	1
**Impairment in Memory Concentration**	0	2	0.49
**Insomnia**	0	2	0.49
**Itchy Scalp**	0	1	1
**Lightheadedness**	0	1	1
**Loss of Appetite**	0	3	0.24
**Low Back Muscle Spasm**	1	0	1
**Low Back Pain/Soreness**	1	0	1
**Nasal Congestion/Runny Nose**	3	2	1
**Nausea / Stomach Discomfort**	2	0	0.49
**Pain**	0	1	1
**Shortness of breath**	0	1	1
**Sore Throat**	1	1	1
**Stye**	0	1	1
**Total**	37	38	1

Supplemental Table S4. Neural Regions of Interest for fMRI Task Analysis

Regions of Interest (ROIs)	Coordinates		
	X	Y	Z
**Right Amygdala**	28	-4	-16
**Left Amygdala**	-28	-4	-16
**Right NAC**	10	6	5
**Left NAC**	-10	6	8
**mOFC**	3	44	-9
**Right dACC**	6	30	24
**Left dACC**	-6	30	24
**Right Ventral Striatum**	26	44	4
**Left Ventral Striatum**	-26	44	4
**Right Insula**	43	−4	17
**Left Insula**	-43	−4	17

Abbreviations: dACC: dorsal Anterior Cingulate Cortex; fMRI: functional Magnetic Resonance Imaging; mOFC: medial Orbito-Frontal Cortex; NAC: Nucleus Accumbens

REFERENCE

Behzadi, Y., Restom, K., Liau, J., & Liu, T. T. (2007). A component based noise correction method (CompCor) for BOLD and perfusion based fMRI. *Neuroimage, 37*(1), 90-101. doi:10.1016/j.neuroimage.2007.04.042
